# Protective effects of vascular endothelial growth factor in cultured brain endothelial cells against hypoglycemia

**DOI:** 10.1007/s11011-015-9659-z

**Published:** 2015-03-13

**Authors:** Fei Zhao, Jiangshan Deng, Xiaoyan Yu, Dawei Li, Hong Shi, Yuwu Zhao

**Affiliations:** 1 Neurologic Department, Shanghai Jiao Tong University Affiliated Sixth People’s Hospital, No.600, Yishan Road, Xuhui District, Shanghai, 200233 China; 2 School of Pharmacy, Shanghai Jiao Tong University, No.800, Dongchuan Road, Minhang District, Shanghai, 200240 China

**Keywords:** Hypoglycemia, Tight junctions, VEGF, Glut-1, Bcl-2

## Abstract

Hypoglycemia is a common and serious problem among patients with type 1 diabetes receiving treatment with insulin. Clinical studies have demonstrated that hypoglycemic edema is involved in the initiation of hypoglycemic brain damage. However, the mechanisms of this edema are poorly understood. Vascular endothelial growth factor (VEGF), a potent regulator of blood vessel function, has been observed an important candidate hormone induced by hypoglycemia to protect neurons by restoring plasma glucose. Whether VEGF has a protective effect against hypoglycemia-induced damage in brain endothelial cells is still unknown. To investigate the effects of hypoglycemia on cerebral microvascular endothelial cells and assess the protective effect of exogenous VEGF on endothelial cells during hypoglycemia, confluent monolayers of the brain endothelial cell line bEnd.3 were treated with normal (5.5 mM glucose), hypoglycemic (0, 0.5, 1 mM glucose) medium or hypoglycemic medium in the presence of VEGF. The results clearly showed that hypoglycemia significantly downregulated the expression of claudin-5 in bEnd.3 cells, without affecting ZO-1 and occludin expression and distribution. Besides, transendothelial permeability significantly increased under hypoglycemic conditions compared to that under control conditions. Moreover, the hypoglycemic medium in presence of VEGF decreased endothelial permeability via the inhibition of claudin-5 degradation and improved hypoglycemia-induced cell toxicity. Furthermore, Glucose transporter-1 (Glut-1) and apoptosis regulator Bcl-2 expression were significantly upregulated. Taken together, hypoglycemia can significantly increase paraendocellular permeability by downregulating claudin-5 expression. We further showed that VEGF protected brain endothelial cells against hypoglycemia by enhancing glucose passage, reducing endothelial cell death, and ameliorating paraendocellular permeability.

## Introduction

Hypoglycemia is one of the most common side effects in the treatment of diabetes, which can lead to recurrent morbidity and sometimes fatality. Glucose is the main energy substrate necessary for normal brain activity. Deficiencies in brain glucose (hypoglycemia) can immediately lead to mild brain dysfunction or even irreversible brain damage (Yun and Ko 2015). Three large clinical trials, Action to Control Cardiovascular Risk in Diabetes (ACCORD), Action in diabetes and Vascular Disease (ADVANCE), and the Veterans Affairs Diabetes Trial, have revealed that episodes of severe hypoglycemia were associated with an increased risk of subsequent mortality and morbidity (Bonds et al. 2010). In general, hypoglycemia occurs commonly in patients with both type 1 and type 2 diabetes. In a prospective survey, 85.3 and 43.6 % of patients with type 1 and type 2 diabetes, respectively, reported experiencing at least one hypoglycemia event over 30 days, while 13.4 and 6.4 %, respectively, reported at least one episode of severe hypoglycemia (Cariou et al. 2014). A recent epidemiological study reported that 84 % of patients with type 2 diabetes experienced at least one hypoglycemic event, and that 42 % of the hypoglycemic episodes were asymptomatic (Wendel et al. 2014). Moreover, recurrent mild/moderate hypoglycemia is more common and may have more serious clinical threats, because it can induce maladaptive responses that obscure the symptoms of hypoglycemia (hypoglycemia unawareness), diminishes counter-regulatory effects to subsequent hypoglycemia, and eventually jeopardize patients’ safety (Cryer 2004). Recent studies demonstrated that recurrent mild/moderate hypoglycemia affects brain function in terms of causing cognitive dysfunction (Schultes et al. 2005), depression and anxiety (Wild et al. 2007). Severe hypoglycemia may result in cognitive impairment, coma, seizures and even death. However, despite being a more severe clinical event, hypoglycemia has received much less attention from medical workers and patients than hyperglycemia.

The blood–brain barrier (BBB), which maintains homeostasis in brain tissues, plays an important role in hypoglycemic brain damage. It is well known that the BBB is maintained primarily by tight junctions (TJs) between brain microvascular endothelial cells (Zlokovic 2008). TJs are composed of transmembrane proteins, including claudins, occludin and junctional adhesion molecule-1 (JAM-1), and peripheral membrane proteins in the zonula occludens family (ZO-1, ZO-2, and others), which prevent the infiltration of substances from the bloodstream into the brain. Therefore, in this study, we attempt to investigate whether hypoglycemia induces cerebral endothelial dysfunction by regulating claudin-5, occluding and ZO-1.

VEGF is an endothelial specific mitogen, a potent mediator of angiogenesis (Medinger and Passweg 2014), and an enhancer of vascular permeability (Brkovic and Sirois 2007). VEGF regulates blood vessel growth, and has been implicated in neuroprotection, neurogenesis, neuronal patterning, glial growth (Wuestefeld et al. 2012), and endothelial cell resistance to death (Byeon et al. 2010; Park et al. 2001). Studies have shown that hypoglycemia dramatically induces VEGF mRNA expression in various cells (Textor et al. 2006; Park et al. 2001). Further, VEGF treatment in ischemic rats significantly improves neurologic recovery (Dzietko et al. 2013). However, the long-term effects of VEGF treatment in hypoglycemic brain endothelial cells are unclear. In the present study, we investigated the protective effects of abluminal application of VEGF during hypoglycemia. Interestingly, long-term abluminal VEGF treatment did not aggravate endothelial permeability.

To elucidate the underlying mechanism regulating BBB permeability during hypoglycemia and the potential role of exogenous VEGF in protecting brain endothelial cells against hypoglycemia, we established an in vitro BBB model using mouse brain microvascular endothelial cells (bEnd.3) which were cultured in normal glucose (5.5 mM glucose) or low glucose (0, 0.5, or 1 mM glucose) for varying times. This was done in the hope of mimicking clinical hypoglycemia conditions, considering that brain glucose concentrations approaches zero when blood glucose concentrations falls below 2 mM (Choi et al. 2001), and within 24 h of hypoglycemic duration was used in many studies (Merino et al. 2014; Sajja et al. 2014).

## Materials and methods

### Cell culture and treatments

Immortalized mouse bEnd.3 cells were purchased from the American Type Culture Collection (ATCC). The cells were seeded on 12-well glass slides for immunofluorescence studies, 12-well Transwell inserts (0.4 μm pore/12 mm diameter, Corning) for endothelial permeability, 24-well plates to analyze cell viability, or in 6-well plates for western blot studies. Cell cultures were incubated in complete Dulbecco’s modified Eagle’s medium (DMEM; Gibco) containing 5.5 mM glucose supplemented with 10 % fetal bovine serum and 1 % penicillin-streptomycin in a humidified incubator at 37 °C in an atmosphere of 5 % CO_2_ and 95 % air. Culture medium was replaced every other day until the bEnd.3 monolayer reached confluence (2–3 d). Cells were used between passages 3 and 15 in all experiments.

For hypoglycemic challenges, confluent bEnd.3 cells were cultured in normal growth medium for 3 d, followed by culturing in normal glucose (5.5 mM glucose) or low glucose (0, 0.5, or 1 mM glucose) for varying times. In some experiments, hypoglycemic cells were treated with various VEGF concentrations for 24 h.

### Western blotting

bEnd.3 cells were lysed in radio immunoprecipitation (RIPA) buffer (50 mM Tris, pH 7.4, 150 mM NaCl, 1 % NP-40, 0.5 % sodium deoxycholate, 0.1 % SDS, and 1 mM EDTA) (Thermo Fisher Scientific, USA), supplemented with 1 % phosphatase inhibitor cocktail and protease inhibitor cocktail (Thermo Fisher Scientific, USA). Protein concentrations were estimated with a BCA protein assay kit (Thermo Fisher Scientific, USA). Equal amounts of protein were loaded and separated by SDS-PAGE gel electrophoresis. Proteins were transferred onto nitrocellulose membranes. Next, the membranes were blocked for 1 h in 5 % non-fat dry skim milk prepared in TBS-T buffer (10 mM Tris–HCl, 150 mM NaCl, and 0.1 % Tween-20), and incubated with primary antibodies targeting claudin-5 (diluted 1:500, Invitrogen), ZO-1 (diluted 1:500, Invitrogen), occludin (diluted 1:500, Invitrogen), or Glut-1 (diluted 1:500, Santa Cruz) in 5 % BSA TBS-T overnight at 4 °C. The blots were then incubated with the respective anti-rabbit or anti-mouse secondary antibodies for 1 h at room temperature (RT). After washing with TBS-T, bands were detected using an infrared scanner (Odyssey, LI-COR Bioscience, Lincoln, NE, USA). β-Actin served as the internal control. Western blots were repeated at least three times for each sample.

### Na-F permeability assay

Endothelial permeability was measured by assessing the transendothelial transport of Na-F (376 Da) as previously described (Dohgu et al. 2004). bEnd.3 cells were grown on Transwell membrane inserts and were exposed to normal glucose, low glucose, or low glucose with 100 ng/ml VEGF for the indicated times. Briefly, 1.5 ml of culture medium was added to the abluminal chamber and 0.5 ml was loaded to the luminal chamber. One hour prior to starting the experiment, 100 μg/ml Na-F (5 μl) in PBS was added to the luminal chamber. After incubation for 60 min, the samples were collected from the abluminal chamber. Na-F flux was determined with a fluorescence multi-well plate reader (Ex (λ) 485 nm; Em (λ) 530 nm). Cell-free samples with no added Na-F served as controls.

### Immunofluorescence

Cultured bEnd.3 cells on glass cover slips were fixed with 4 % paraformaldehyde for 30 min and permeabilized with 0.2 % Triton X-100 in PBS for 15 min at RT. After washing with PBS three times, the cells were blocked with 5 % goat serum in PBS for 30 min. Subsequently, the cells were incubated with primary antibodies against claudin-5 (1:100, Invitrogen), ZO-1 (1:100, Invitrogen), and occludin (1:100, Invitrogen) at RT for 2 h, followed by washing four times with PBS. Cells were incubated with secondary antibodies (anti-mouse or anti-rabbit Alexa Fluor 488 (1:1000)) for 1 h at RT. Cells stained without primary antibodies served as negative controls. Nuclei were stained with 1 μg/ml DAPI for 5 min. The cells were washed thoroughly with PBS and mounted in fluorescent mounting medium for analysis under a confocal microscope.

### Lactate dehydrogenase (LDH) assay

Following exposure to normal or hypoglycemic media, with or without 100 ng/ml VEGF, supernatants were collected, and LDH release was determined using an LDH assay kit (Beyotime, China) according to the manufacturer’s guidelines.

### Statistical analyses

All data were expressed as the mean ± SEM. An ANOVA was used to analyze the data, and a *P*-value of less than 0.05 was considered statistically significant.

## Results

### Effect of hypoglycemia on endothelial permeability

**Fig. 1:**
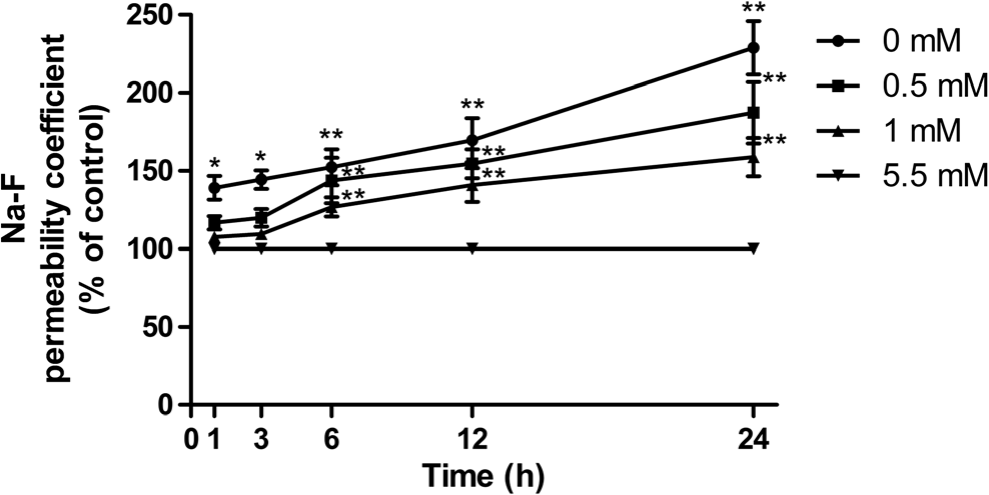
Time-course of the changes in paracellular permeability of bEnd.3 cells after exposure to various concentrations of hypoglycemia during a 24-h period. bEnd.3 cells were plated in a monolayer with Na-F applied. Na-F was evaluated following treatment with 5.5 mM (control) or 0, 0.5, or 1 mM over 1, 3, 6, 12, and 24 h. Data are expressed as the percentage of the time-matched control value. Data are expressed as the mean ± SEM. *N* = 4. ^*^ *P* < 0.05, ^**^ *P* < 0.01

To determine the effect of hypoglycemia on endothelial permeability, confluent bEnd.3 cell monolayers were incubated with varying glucose concentrations for 0 to 24 h, and the Na-F permeability was analyzed using a Costar Transwell system (Corning, USA) based on a previous protocol (Dohgu et al. 2004). As shown in Fig. 1, Compared with 5.5 mM glucose, 0 mM glucose increased the Na-F permeability by 28.1, 30.8, 34.3, 41.1, and 56.3 % at 1, 3, 6, 12, and 24 h, respectively. Compared with 5.5 mM glucose, 0.5 and 1 mM glucose increased the Na-F permeability at 6, 12, and 24 h (by 30.5, 35.3, and 46.6 %, respectively; 21.2, 29.1, and 37 %, respectively) when compared with 5.5 mM glucose. 0 mM glucose caused significant increase in endothelial permeability after 1 h, while 0.5 and 1 mM glucose increased permeability significantly after 6 h. Besides, lower glucose concentrations led to a greater increase in paracellular permeability than higher glucose concentrations after 6, 12 or 24 h treatment. Hypoglycemia increased brain endothelial permeability time- dependently and glucose concentration-dependently.

### Effect of hypoglycemia on the expression of claudin-5, ZO-1, and occludin in bEnd.3 cells

**Fig. 2:**
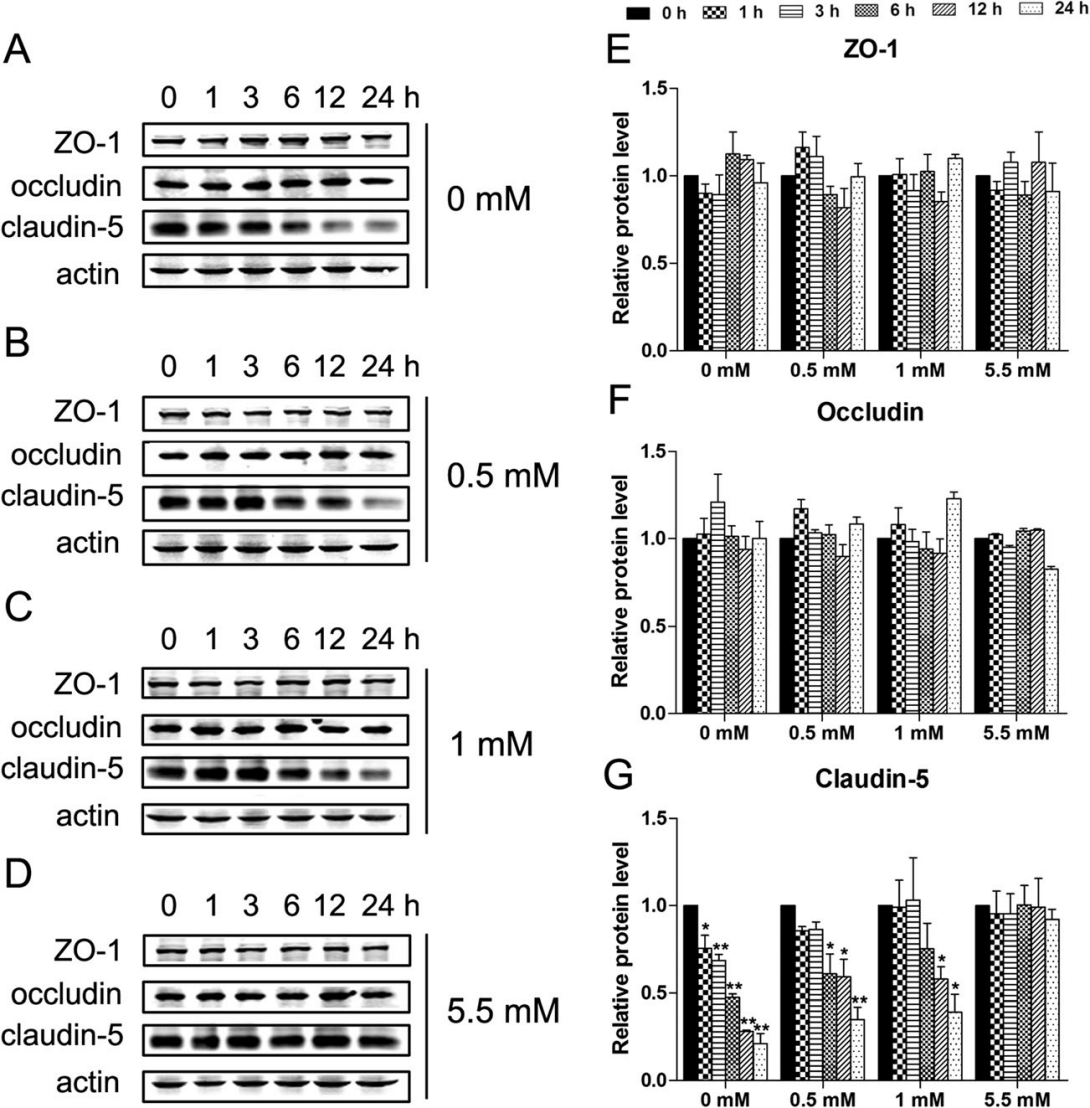
Effects of hypoglycemia on TJ proteins in bEnd.3 cells. Confluent bEnd.3 monolayers were exposed to 0 mM (**a**), 0.5 mM (**b**), 1 mM (**c**), or 5.5 mM (**d**, control) glucose for 1, 3, 6, 12, or 24 h. Representative blots for ZO-1, occludin, claudin-5, and β-actin are shown. Summary plots of ZO-1 (**e**), occludin (**f**), and claudin-5 (**g**) expression, including densitometric reading of the corresponding protein blots, are shown. Data are expressed as the mean ± SEM. *N* = 3. ^*^ *P* < 0.05, ^**^ *P* < 0.01

**Fig. 3:**
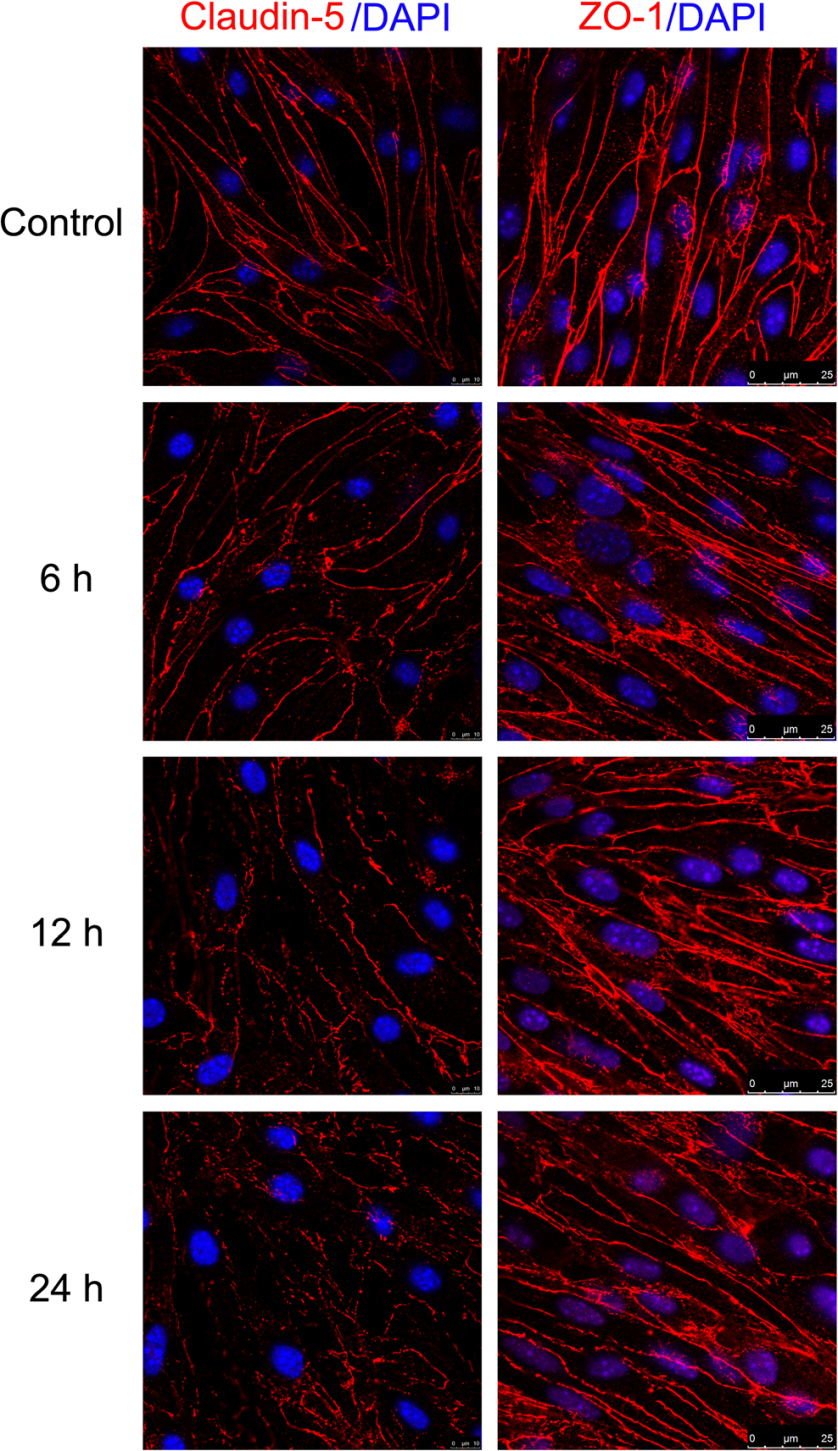
Effects of hypoglycemia on the expression and distribution of claudin-5 and ZO-1 in bEnd.3 cells. Confluent bEnd.3 cell monolayers were exposed to 0.5 mM conditioned medium for 0 (control), 6, 12, and 24 h. Cultures were then stained with claudin-5 (*red*), ZO-1 (*red*) and DAPI (nuclear, *blue*) to examine protein expression, distribution, and cell morphology changes. *N* = 3

Tight junctions integrity is a hallmark of brain edema progression in pathological conditions such as stroke (Jiao et al. 2011). It has been shown that TJ proteins, including occludin, ZO-1, and claudin-5, have an important role in the integrity of the BBB (Sandoval and Witt 2008). To investigate whether these proteins have a major role in hypoglycemia, western blot and immunofluorescence analyses were performed to detect the protein expression levels. As shown in Fig. 2, western blot analysis revealed that hypoglycemia induced a time- and glucose dose-dependent decrease in claudin-5 protein levels. However, hypoglycemia did not significantly change ZO-1 and occludin expression compared to the control (5.5 mM glucose). Furthermore, as shown in Fig. 3, immunofluorescence imaging confirmed that hypoglycemia did not alter the localization of claudin-5, ZO-1, and occludin after 6, 12, or 24 h treatment, although occludin is displayed diffuse cytoplasmic localization in bEnd.3 (data not shown). These results were consistent with the observed hypoglycemia-induced permeability increase in the endothelial monolayer. These data suggested that disruption of claudin-5 may increase BBB permeability.

### Exogenous VEGF ameliorates hypoglycemia-induced brain endothelial cells dysfunction

**Fig. 4:**
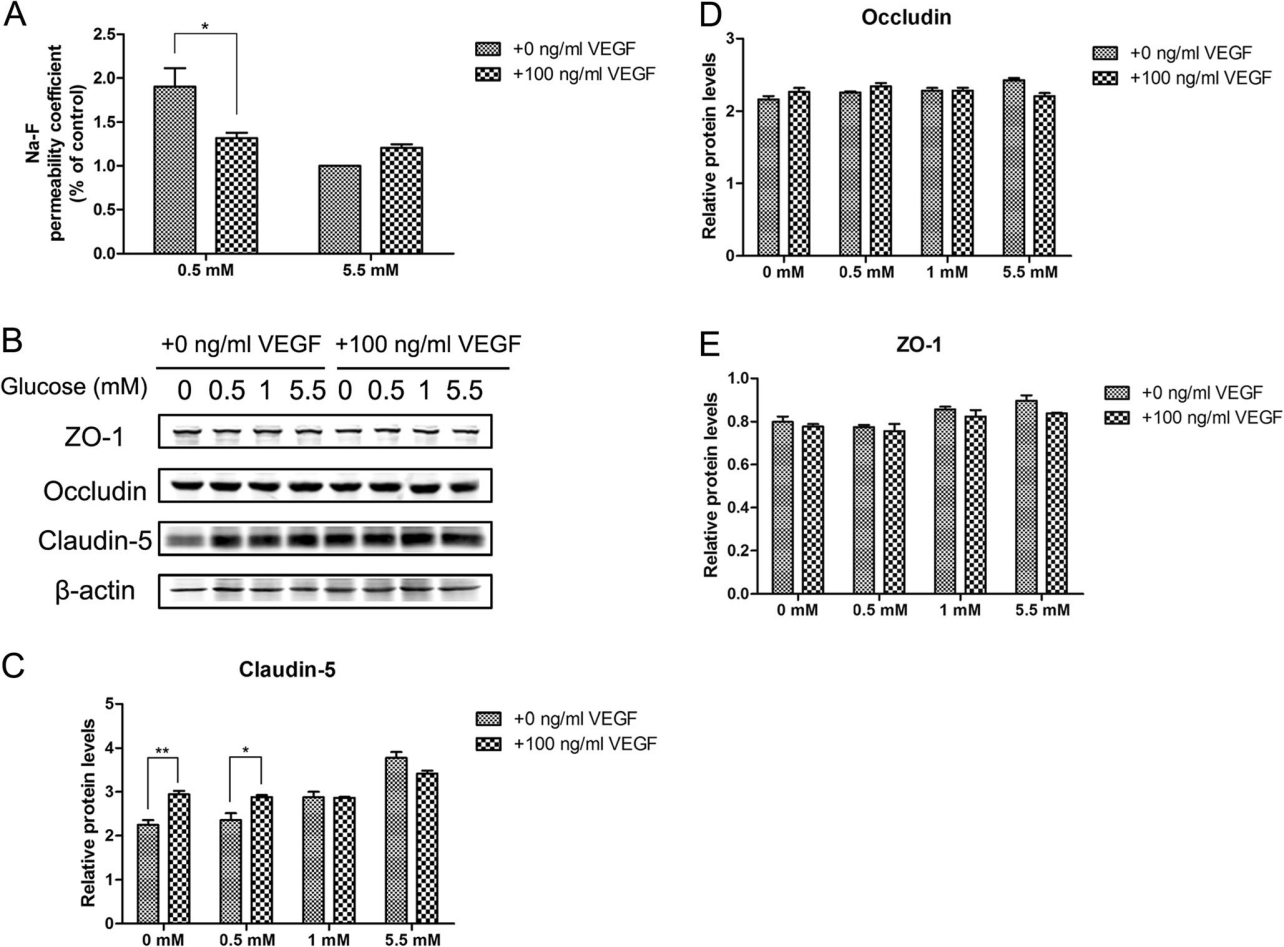
Effects of VEGF on hypoglycemia-induced paracellular permeability and TJ proteins. (**a**) Effect of VEGF (100 ng/ml) on cell permeability of cells exposed to 0.5 and 5.5 mM glucose for 24 h. (**b**) Effects of VEGF on TJ protein levels during hypoglycemia for 24 h in bEnd.3 cells. Representative TJ Western blots are shown following treatment with 0.5 or 5.5 mM glucose for 24 h with the presence of 100 ng/ml VEGF. Summary plots of claudin-5 (**c**), occludin (**d**), and ZO-1 (**e**) are shown following densitometric analysis of the corresponding protein blots. Data are expressed as the mean ± SEM. *N* = 3. ^*^ *P* < 0.05, ^**^ *P* < 0.01

Since TJ-mediated regulation of endothelial permeability is vital to BBB integrity, we assessed the effect of exogenous VEGF (100 ng/ml) on permeability in bEnd.3 cells under hypoglycemic conditions by measuring the ability of Na-F to cross the cell monolayer 24 h after 0.5 mM glucose treatment. As shown in Fig. 4a, a 24 h exposure to 0.5 mM glucose in presence of VEGF decreased the Na-F permeability by 30.8 % compared with 0.5 mM glucose, suggesting the presence of VEGF significantly decreased the enhanced endothelial permeability caused by hypoglycemia.

In order to investigate whether VEGF modulates the disruption of TJ proteins in hypoglycemic conditions, we assessed the expression levels of claudin-5, occludin, and ZO-1. Figure 4b and c showed that the decrease in claudin-5 was improved under hypoglycemia in presence of VEGF. Western blot analysis revealed no change in the expression of ZO-1 and occludin in bEnd.3 cells treated with VEGF. Overall, these results suggested that VEGF has a beneficial effect on endothelial cells during hypoglycemic stress.

### Effect of exogenous VEGF on survival of bEnd.3 cells under hypoglycemic conditions

**Fig. 5:**
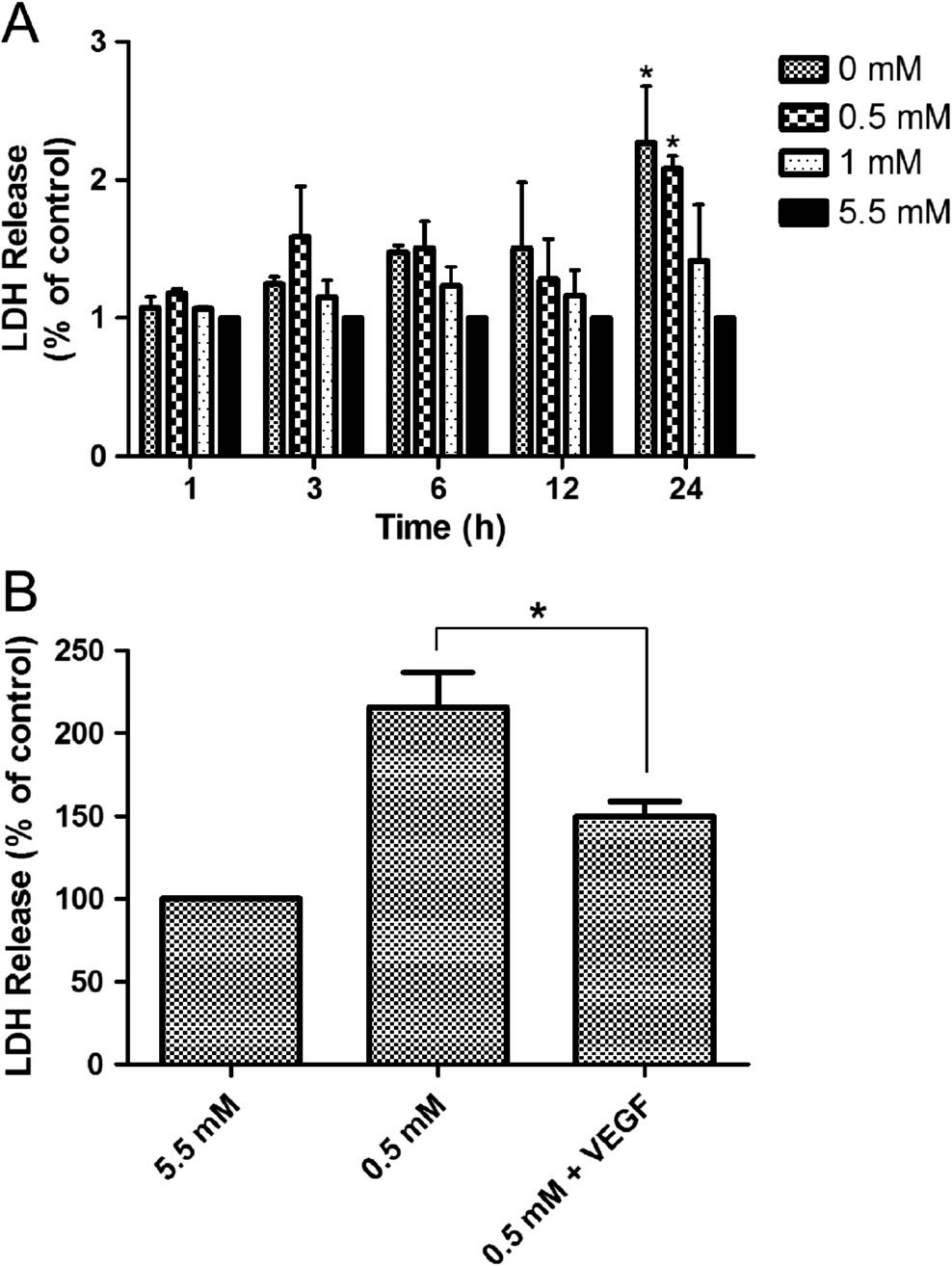
Effects of hypoglycemia and VEGF treatment on cell viability. (**a**) Cells were maintained in 5.5 mM glucose (control) or 0, 0.5 and 1 mM glucose for the indicated times. The results are given as a percent relative to the control. (**b**) VEGF (100 ng/mL) was added to cultures at the onset of exposure to 0.5 and 5.5 mM (control) mM glucose medium for 24 h. Cell viability was determined by the LDH assay. Data are expressed as mean ± SEM. *N* = 3. ^*^ *P* < 0.05, ^**^ *P* < 0.01

Endothelial cell death can augment BBB permeability (Engelhardt et al. 2014). Application of VEGF enhances cell survival and protects neurons against ischemic injury (Nishijima et al. 2007). We firstly assessed whether long-term hypoglycemia can induce bEnd.3 cell damage. Figure 5a showed that bEnd.3 cell viability decreased progressively after 24 h incubation with 0 and 0.5 mM glucose medium. To further examine the effect of VEGF on hypoglycemia-induced cell death, bEnd.3 cells were exposed to hypoglycemia in presence of VEGF (100 ng/ml). Figure 5b showed that a 24 h exposure to 0.5 mM glucose in presence of VEGF decreased cell death by 30.5 % compared with 0.5 mM glucose, suggesting the presence of VEGF significantly reduced cell death from hypoglycemia.

### Hypoglycemia in presence of VEGF highly induces the expression of Glut-1 and Bcl-2 in endothelial cells

**Fig. 6:**
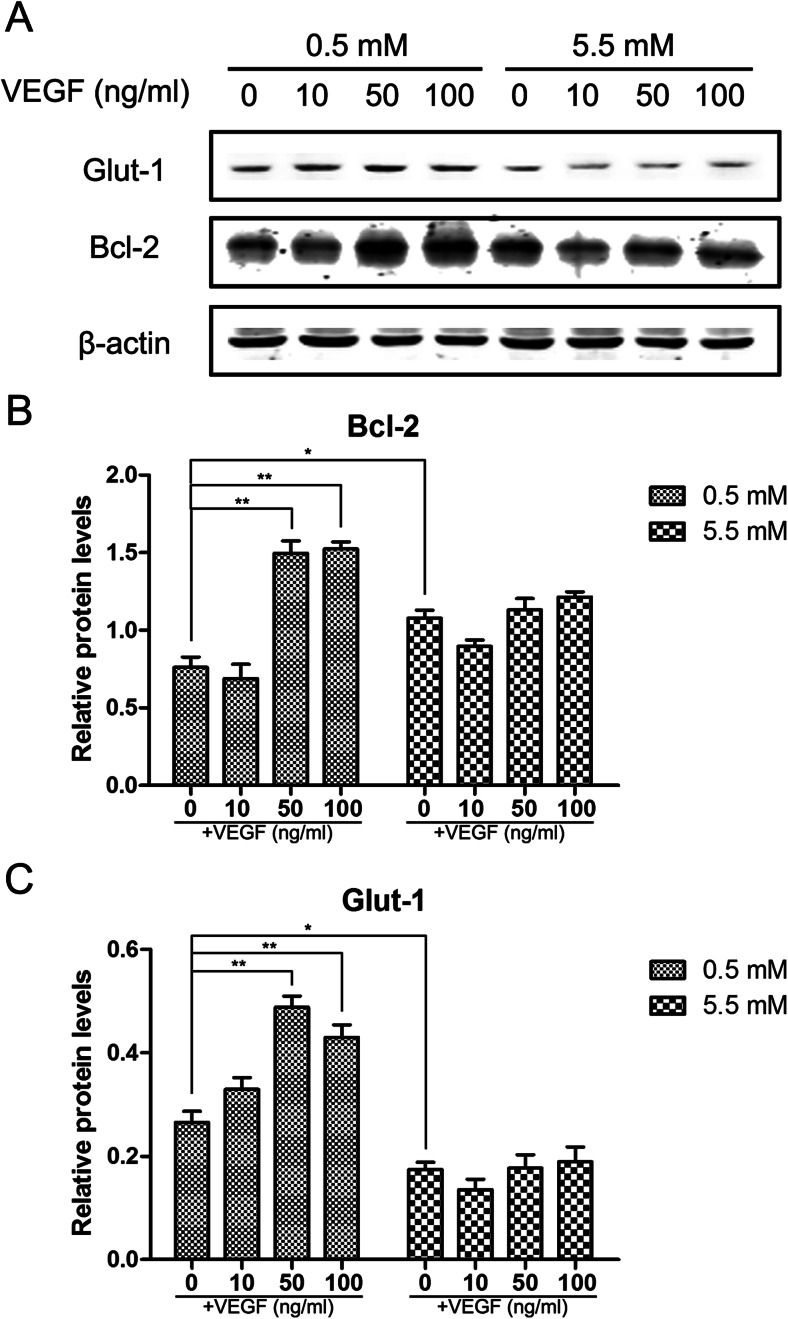
Dose-dependent effects of VEGF on the Glut-1 and Bcl-2 during hypoglycemia in bEnd.3 cells. Confluent bEnd.3 cells were treated with 5.5 mM (control) glucose and 0.5 mM glucose for 24 h in the absence or presence of different concentrations of VEGF (0, 10, 50, and 100 ng/mL). (**a**) Representative Western blots of Glut-1 and Bcl-2 with β-actin as a protein loading control. Summary plots of Bcl-2 (**b**) and Glut-1 (**c**) with densitometric analyses of the corresponding protein blots. Data are expressed as mean ± SEM. *N* = 3. ^*^ *P* < 0.05, ^**^ *P* < 0.01

Apoptosis of brain endothelial cells following injury is related to downregulation of constitutively expressed anti-apoptotic factors, which is known to further promote BBB breakdown (Nag et al. 2005). To understand the underlying mechanisms of VEGF-mediated protection against hypoglycemia, we analyzed Glut-1 and Bcl-2 protein levels by western blot in response to VEGF during hypoglycemia. We found that Glut-1 and Bcl-2 levels in bEnd.3 cells increased in a dose-dependent manner following 24-h VEGF treatment under hypoglycemic conditions. No significant changes in Glut-1 and Bcl-2 were observed in response to VEGF stimulation when cells were grown in normal glucose medium (Fig. 6). These findings suggested that VEGF upregulates Glut-1 and Bcl-2 protein expression under hypoglycemic conditions. These data supported a mechanism in which VEGF protects cerebral endothelial cells against hypoglycemia by decreasing hypoglycemia-induced apoptosis and promoting the transfer of glucose.

## Discussion

The loss of TJs among adjacent endothelial cells that regulate endothelial permeability (Liu et al. 2012) is the main factor contributing to brain edema. Many studies have demonstrated that claudins, but not occludins, are the major proteins involved in TJ formation (Jia et al. 2014; Haseloff et al. 2014), which is essential for maintaining BBB structure. For example, claudin-5 deletion resulted in a size-selective increase in permeability (Stamatovic et al. 2008). Further, claudin-5-deficient mice also displayed an altered BBB with higher permeability (Luissint et al. 2012). It has been shown that hypoxia causes degradation of occludin, ZO-1, ZO-2, and claudin-3 (González-Mariscal et al. 2011; Witt et al. 2003). We hypothesized that altered expression of TJ proteins might contribute to increased paraendothelial permeability during hypoglycemia. In the present study, we induced hypoglycemic injury in brain endothelial cells to evaluate the effect of hypoglycemia on TJ proteins. Our data indicated that hypoglycemia disrupted claudin-5 expression, without affecting the translocation of claudin-5, and increased paraendothelial permeability. The potential mechanisms regulating the change in claudin-5 expression is still unclear, although we speculate that it is related to glucose depletion during hypoglycemia.

Previous studies have confirmed that hypoglycemia induces rapid upregulation of VEGF expression, and that VEGF mediates the rapid adaptation of the brain to hypoglycemia (Oltmanns et al. 2008). While several studies have demonstrated that VEGF causes increased BBB permeability (Argaw et al. 2012; Feng et al. 2011), others have shown that administration of VEGF intracerebroventricularly protects the brain against focal ischemia without increasing BBB permeability (Kaya et al. 2005). The specific mechanism of the VEGF-induced increase in BBB permeability is controversial. Plate suggested that VEGF plays a role in the protection and maintenance of endothelial cells (Plate 1999). To assess whether application of VEGF has a protective effect on endothelial cells during hypoglycemia, we detected the paraendothelial permeability, TJ-associated protein expression, and endothelial cell viability. We found that long-term VEGF treatment decreased endothelial permeability following hypoglycemic injury. These results appeared to contradict data that suggests VEGF plays a vital role in brain edema. However, analysis of the effect of VEGF on barrier dysfunction in vitro revealed that VEGF induces transient and reversible permeability in cultured brain endothelial cells (Senger et al. 1983). Our findings suggest that VEGF may prevent BBB disruption during hypoglycemia by inhibiting the degradation of claudin-5. However, the exact mechanisms of VEGF-induced claudin-5 expression are not completely understood.

VEGF, a potent mediator of angiogenesis, is a candidate hormone for facilitating glucose passage across the BBB under critical conditions. VEGF has also been shown to enhance glucose transport by increasing Glut-1 expression, which is an ideal target to supply much needed glucose required to meet cellular metabolic requirements (Sone et al. 2000). During hypoglycemia, Glut-1 gene and protein expression levels increase in vivo (Sahin et al. 2013), thereby increasing BBB sugar transport capacity. Thus, VEGF may protect the glucose supply in the brain by increasing the protein expression of Glut-1. In the present study, Glut-1 protein levels were significantly increased in hypoglycemic cells treated with VEGF compared to untreated hypoglycemic cells. Moreover, several animal studies have suggested that VEGF reduces cellular damage in ischemia and hypoxia injury, and that Bcl-2, an anti-apoptotic protein, can be induced by VEGF (Lladó et al. 2013). We found the Bcl-2 protein levels in VEGF-treated hypoglycemic cells were significantly increase compared to untreated hypoglycemic cells. The overall increase of Bcl-2 protein was observed under hypoglycemic conditions in the presence of VEGF, suggesting that VEGF is an endothelial cell survival factor. Further, Akt activation by PI3-kinase has been shown to cause increases in VEGF expression in some cell types (Pore et al. 2004; Liu et al. 2006), and has been shown to protect against cell death (Byeon et al. 2010). In brain microendothelial cells, Akt inhibits apoptosis and enhances cell survival (Zhou et al. 2013). Thus, the protective effect of VEGF against hypoglycemia is mediated in part by the PI-3 K/Akt pathway. This was confirmed by an LDH assay that was used to measure cell death. No significant changes in Glut-1 and Bcl-2 were observed in response to VEGF stimulation when endothelial cells were grown in a normal glucose medium.

In summary, the present study demonstrated that hypoglycemia disrupts the BBB by reducing claudin-5 expression and increasing endothelial cell death, thereby increasing endothelial permeability. VEGF can protect brain endothelial cells against hypoglycemic injury by increasing Glut 1, Bcl-2, and claudin-5 expression to promote glucose transport, reduce cell death, and prevent BBB disruption. Although debates continue over the role of VEGF in hypoglycemia and this study has several limitations, our findings may provide the basic data required for further study of hypoglycemia.

## References

[CR1] Argaw AT, Asp L, Zhang J, Navrazhina K, Pham T, Mariani JN, Mahase S, Dutta DJ, Seto J, Kramer EG, Ferrara N, Sofroniew MV, John GR (2012). Astrocyte-derived VEGF-A drives blood–brain barrier disruption in CNS inflammatory disease. J Clin Invest.

[CR2] Bonds DE, Miller ME, Bergenstal RM, Buse JB, Byington RP, Cutler JA, Dudl RJ, Ismail-Beigi F, Kimel AR, Hoogwerf B, Horowitz KR, Savage PJ, Seaquist ER, Simmons DL, Sivitz WL, Speril-Hollen JM, Sweeney ME (2010). The association between symptomatic, severe hypoglycemia and mortality in type 2 diabetes: retrospective epidemiological analysis of the ACCORD study. BMJ.

[CR3] Brkovic A, Sirois MG (2007). Vascular permeability induced by VEGF family members in vivo: role of endogenous PAF and NO synthesis. J Cell Biochem.

[CR4] Byeon SH, Lee SC, Choi SH, Lee HK, Lee JH, Chu YK, Kwon OW (2010). Vascular endothelial growth factor as an autocrine survival factor for retinal pigment epithelial cells under oxidative via the VEGF-R2/PI3K/Akt. Invest Ophthalmol Vis Sci.

[CR5] Cariou B, Fontaine P, Eschwege E, Lièvre M, Gouet D, Madani S, Lavigne S, Charbonnel B (2014). Frequency and predictors of confirmed hypoglycemia in type 1 and insulin-treated type 2 diabetes mellitus patients in a real-life setting: Results from the DIALOG study. Diabetes Metab.

[CR6] Choi IY, Lee SP, Kim SG, Gruetter R (2001). In vivo measurements of brain glucose transport using the reversible Michaelis-Menten model and simultaneous measurements of cerebral blood flow changes during hypoglycemia. J Cereb Blood Flow Metab.

[CR7] Cryer PE (2004). Diverse causes of hypoglycemia-associated autonomic failure in diabetes. N Engl J Med.

[CR8] Dohgu S, Yamauchi A, Nakagawa S, Takata F, Kai M, Egawa T, Naito M, Tsuruo T, Sawada Y, Niwa M, Kataoka Y (2004). Nitric oxide mediates cyclosporine-induced impairment of the blood–brain barrier in cocultures of mouse brain endothelial cells and rat astrocytes. Eur J Pharmacol.

[CR9] Dzietko M, Derugin N, Wendland MF, Vexler ZS, Ferriero DM (2013). Delayed VEGF treatment enhances angiogenesis and recovery after neonatal focal rodent stroke. Transl Stroke Res.

[CR10] Engelhardt S, Patkar S, Ogunshola OO (2014). Cell-specific blood–brain barrier regulation in health and disease: a focus on hypoxia. Br J Pharmacol.

[CR11] Feng S, Huang Y, Chen Z (2011). Does VEGF secreted by leukemic cells increase the permeability of blood–brain barrier by disrupting tight-junction proteins in central nervous system leukemia?. Med Hypotheses.

[CR12] González-Mariscal L, Quirós M, Díaz-Coránguez M (2011). ZO proteins and redox-dependent processes. Antioxid Redox Signal.

[CR13] Haseloff RF, Dithmer S, Winkler L, Wolburg H, Blasig IE (2014). Transmembrane proteins of the tight junctions at the blood–brain barrier: structural and functional aspects. Semin Cell Dev Biol.

[CR14] Jia W, Lu R, Martin TA, Jiang WG (2014). The role of claudin-5 in blood–brain barrier (BBB) and brain metastases (review). Mol Med Rep.

[CR15] Jiao H, Wang Z, Liu Y, Wang P, Xue Y (2011). Specific role tight junction proteins claudin-5, occludin, and ZO-1 of the blood–brain barrier in a focal cerebral ischemic insult. J Mol Neurosci.

[CR16] Kaya D, Gürsoy-Ozdemir Y, Yemisci M, Tuncer N, Aktan S, Dalkara T (2005). VEGF protects brain against focal ischemia without increasing blood–brain permeability when administered intracerebroventricularly. J Cereb Blood Flow Metab.

[CR17] Liu LZ, Hu XW, Xia C, He J, Zhou Q, Shi X, Fang J, Jiang BH (2006). Reactive oxygen species regulate epidermal growth factor-induced vascular endothelial growth factor and hypoxia-inducible factor-1α expression through activation of AKT and P70S6K1 in human ovarian cancer cells. Free Radic Biol Med.

[CR18] Liu WY, Wang ZB, Zhang LC, Wei X, Li L (2012). Tight junction in blood–brain barrier: an overview of structure, regulation, and regulator substances. CNS Neurosci Ther.

[CR19] Lladó J, Tolosa L, Olmos G (2013). Cellular and molecular mechanisms involved in the neuroprotective effects of VEGF on motoneurons. Front Cell Neurosci.

[CR20] Luissint AC, Federici C, Guillonneau F, Chrétien F, Camoin L, Glacial F, Ganeshamoorthy K, Couraud PO (2012). Guanine nucleotide-binding protein Gαi2: a new partner of claudin-5 that regulates tight junction integrity in human brain endothelial cells. J Cereb Blood Flow Metab.

[CR21] Medinger M, Passweg J (2014). Angiogenesis in myeloproliferative neoplasms, new markers and future directions. Memo.

[CR22] Merino JJ, Roncero C, Oset-Gasque MJ, Naddaf A, González MP (2014). Antioxidant and protective mechanisms against hypoxia and hypoglycemia in cortical neurons in vitro. Int J Mol Sci.

[CR23] Nag S, Papneja T, Venugopalan R, Stewart DJ (2005). Increased angiopoietin2 expression is associated with endothelial apoptosis and blood–brain barrier breakdown. Lab Invest.

[CR24] Nishijima K, Ng YS, Zhong L, Bradley J, Schubert W, Jo N, Akita J, Samuelsson SJ, Robinson GS, Adamis AP, Shima DT (2007). Vascular endothelial growth factor-A is a survival factor for retinal neurons and a critical neuroprotectant during the adaptive response to ischemic injury. Am J Pathol.

[CR25] Oltmanns KM, Melchert UH, Scholand-Engler HG, Schultes B, Schweiger U, Peters A (2008). Divergent effects of hyper- and hypoglycemia on circulating vascular endothelial growth factor in humans. Metabolism.

[CR26] Park SH, Kim KW, Lee YS, Baek JH, Kim MS, Lee YM, Lee MS, Kim YJ (2001). Hypoglycemia-induced VEGF expression is mediated by intracellular Ca2+ and protein kinase C signaling pathway in HepG2 human hepatoblastoma cells. Int J Mol Med.

[CR27] Plate KH (1999). Mechanisms of angiogenesis in the brain. J Neuropathol Exp Neurol.

[CR28] Pore N, Liu S, Shu HK, Li B, Haas-Kogan D, Stokoe D, Milanini-Mongiat J, Pages G, O'Rourke DM, Bernhard E, Maity A (2004). Mol Biol Cell.

[CR29] Sahin K, Tuzcu M, Orhan C, Ali S, Sahin N, Gencoglu H, Ozkan Y, Hayirli A, Gozel N, Komorowski JR (2013). Chromium modulates expressions of neuronal plasticity markers and glial fibrillary acidic protein in hypoglycemia-induced brain injury. Life Sci.

[CR30] Sajja RK, Prasad S, Cucullo L (2014). Impact of altered glycaemia on blood–brain barrier endothelium: an in vitro study using the hCMEC/D3 cell line. Fluids Barriers CNS.

[CR31] Sandoval KE, Witt KA (2008). Blood–brain barrier tight junction permeability and ischemic stroke. Neurobiol Dis.

[CR32] Schultes B, Kern W, Oltmanns K, Peters A, Gais S, Fehm HL, Born J (2005). Differential adaption of neurocognitive brain functions to recurrent hypoglycemia in healthy men. Psychoneuroendocrinology.

[CR33] Senger DR, Galli SJ, Dvorak AM, Perruzzi CA, Harvey VS, Dvorak HF (1983). Tumor cells secrete a vascular permeability factor that promotes accumulation of ascites fluid. Science.

[CR34] Sone H, Deo BK, Kumagai AK (2000). Enhancement of glucose transport by vascular endothelial growth factor in retinal endothelial cells. Invest Ophthalmol Vis Sci.

[CR35] Stamatovic SM, Keep RF, Andjelkovic AV (2008). Brain endothelial cell-cell junctions: how to “open” the blood brain barrier. Curr Neuropharmacol.

[CR36] Textor B, Sator-Schmitt M, Richter KH, Angel P, Schorpp-Kistner M (2006). c-Jun and JunB are essential for hypoglycemia-mediated VEGF induction. Ann N Y Acad Sci.

[CR37] Wendel CS, Fotieo GG, Shah JH, Felicetta J, Curtis BH, Murata GH (2014). Incidence of non-severe hypoglycemia and intensity of treatment among veterans with type 2 diabetes in the U.S.A.: a prospective observational study. Diabet Med.

[CR38] Wild D, von Maltzahn R, Brohan E, Christensen T, Clauson P, Gonder-Frederick L (2007). A critical review of the literature on fear of hypoglycemia in diabetes: implications for diabetes management and patient education. Patient Educ Couns.

[CR39] Witt KA, Mark KS, Hom S, Davis TP (2003). Effects of hypoxia-reoxygenation on rat blood–brain barrier permeability and tight junctional protein expression. Am J Physiol Heart Circ Physiol.

[CR40] Wuestefeld R, Chen J, Meller K, Brand-Saberi B, Theiss C (2012). Impact of vegf on astrocytes: analysis of gap junctional intercellular communication, proliferation, and motility. Glia.

[CR41] Yun JS, Ko SH (2015). Avoiding or coping with severe hypoglycemia in patients with type 2 diabetes. Korean J Intern Med.

[CR42] Zhou HG, Liu L, Zhang Y, Huang YY, Tao YH, Zhang S, Su JJ, Tang YP, Guo ZL, Hu RM, Dong Q (2013). Glutathione prevents free fatty acids-induced oxidative stress and apoptosis in human brain vascular endothelial cells through Akt pathway. CNS Neurosci Ther.

[CR43] Zlokovic BV (2008). The blood–brain barrier in health and chronic neurodegenerative disorders. Neuron.

